# Sound Insulation Performance of Composite Double Sandwich Panels with Periodic Arrays of Shunted Piezoelectric Patches

**DOI:** 10.3390/ma15020490

**Published:** 2022-01-10

**Authors:** Shande Li, Di Xu, Xiaoxun Wu, Renjie Jiang, Geman Shi, Zhifu Zhang

**Affiliations:** 1 State Key Laboratory of Digital Manufacturing Equipment and Technology, School of Mechanical Science and Engineering, Huazhong University of Science and Technology, Wuhan 430074, China; lishande@hust.edu.cn (S.L.); ak9962@163.com (D.X.); wxxaug@163.com (X.W.); jrj6320@163.com (R.J.); im1826076599@163.com (G.S.); 2 Automotive NVH Research Center, Hubei Institute of Specialty Vehicle, Suizhou 441300, China

**Keywords:** broadband sound insulation, honeycomb, sandwich composites, shunted piezoelectric patches

## Abstract

The existing sandwich structure of the aircraft cabin demonstrates a good sound insulation effect in medium and high frequency bands, but poor in the low frequency band. Therefore, we propose an infinite new lightweight broadband noise control structure and study its sound transmission loss (STL). The structure is an orthogonally rib-stiffened honeycomb double sandwich structure with periodic arrays of shunted piezoelectric patches, and demonstrates lighter mass and better strength than the existing sandwich structure. The structure is equivalent according to Hoff’s equal stiffness theory and the effective medium (EM) method. Using the virtual work principle for a periodic element, two infinite sets of coupled equations are obtained. They are solved by truncating them in a finite range until the solution converges. The correctness and validity of the model are verified by using simulation results and theoretical predictions. Eventually, a further study is performed on the factors influencing the STL. All the results demonstrate that the STL in low-frequency can be improved by the structure, and the sound insulation bandwidth is significantly broadened by adding shunted piezoelectric patches. The structure can provide a new idea for the design of broadband sound insulation.

## 1. Introduction

The classical sandwich structure is composed of two parallel plates and reinforced by a group of spatially periodic rib-stiffeners, which are widely used in the fields of spaceflight and aviation [1,2,3,4,5,6]. At present, many scholars have studied the acoustic characteristics of classical sandwich structures. Mead [7] found that the harmonic flexural wave propagating freely in an infinite periodically supported beam should be regarded as a wave group with components of different phase velocity, direction, and wavelength. Mace [6] derived a response expression of an infinite fluid loaded stiffened plate to a general excitation. Xin and Lu [8] quantified the influence of different factors, such as different propagation paths, the spacing of stiffeners, and incident angles, on the transmission of a wave across an infinite fluid-loaded sandwich structure.

Honeycomb sandwich plates have the advantages of light weight, good strength, and high stiffness. At present, many scholars have carried out extensive research on the dynamic and static analysis of honeycomb sandwich structures. Kelsey et al. [9] deduced the theoretical expressions of the shear modulus of honeycomb sandwich cores. Kobayashi et al. [10] studied the elastoplastic bending behavior of honeycomb sandwich panels. Kunimo et al. [11,12] studied the buffer characteristics of bare honeycomb cores under lateral crushing loads using experimental and theoretical methods respectively. Due to the complexity of such structures, it is necessary to simplify the structure to a certain extent. There are three common equivalent methods which are the equivalent-plate theory, the honeycomb-plate theory, and the sandwich theory. However, there are few structures that combine the classical sandwich structure with the honeycomb sandwich plate.

Due to the advantages of honeycomb sandwich plates, this paper attempts to combine the classical sandwich structure with it to explore whether there is a better sound absorption effect.

Although the existing sandwich structure has a good sound insulation effect in middle and high frequency bands, the STL in low frequency is still not improved. To improve the sound insulation effect of low frequency, it is necessary to increase the thickness of cavity or the mass of plate, which will increase the mass of aircraft, which is not feasible. Forward [13] first proposed the piezoelectric shunt technique, and Hagood and Von Flotow [14] found that a piezoelectric patch connected with a resistive-inductive circuit can reduce vibration. Reducing structural noise transmission and radiation by utilizing the single piezoelectric shunt is possible, which is addressed by some work undertaken more than ten years ago [15,16,17,18]. In recent years, periodic piezoelectric shunts have attracted extensive attention in the field of sound control. Zhang et al. [19] developed EM method for the calculation of STL and found that the metamaterial plate with shunted piezoelectric patches has much higher STL than the unshunted case in the coincidence region and mass-law region of sound transmission. Casadei et al. [20] studied the application of periodic arrays of shunted piezoelectric patches in controlling the radiated noise of a finite plate in a closed cavity and found that the associated noise and vibration are significantly attenuated. Therefore, in this work, the periodic arrays of shunted piezoelectric patches are introduced into the orthogonally rib-stiffened sandwich structure to improve the low-frequency STL. Based on the use of a single shunted piezoelectric patch for structural stabilization and wave cancellation at a single frequency, extensions have been developed to design more complex shunting circuits to extend the effectiveness over broader frequency bands, using, e.g., multimodal shunts [21,22,23,24,25]. Bricault [26] found that when several modes of a square aluminum plate are damped simultaneously using a piezoelectric patch shunted with a negative capacitor circuit, the low-frequency acoustic radiation of the plate is reduced.

With excellent mechanical properties, composite materials have been applied in the field of acoustics by many scholars. For example, Anish et al. [27] used improved higher order shear deformation theory (IHSDT) to investigate the influence of additional mass and openings on the free vibration analysis of laminated composite sandwich skew plates. Talebitoti and Zarastvand [28] analytically modeled the aerospace composite structure and predicted the acoustic transmission of the infinitely long, doubly curved shell. In addition, they found that current structures with curvature showed better acoustic and mechanical properties, especially at lower frequencies.

On the basis of the foregoing, we develop a new lightweight broadband noise control composite structure which is an orthogonally rib-stiffened honeycomb double sandwich structure with periodic arrays of shunted piezoelectric patches. The validation of the theoretical model is demonstrated by comparing the theoretical results with the results of the finite element simulation software (COMSOL), and the validity of the theoretical model is verified by comparing the sound transmission loss of the structure with and without the shunted piezoelectric patches. Based on the existing classical orthogonally rib-stiffened sandwich structure with good sound insulation in the middle and high frequency bands, the characteristics of light weight and excellent mechanical properties of honeycomb sandwich panels are combined. The periodic arrays of shunted piezoelectric patches are also introduced to improve the sound insulation effect at low frequency. This study is expected to improve the design of the traditional sandwich structure and its sound insulation effect.

## 2. Theoretical Modelling

### 2.1. Description of the Research Structure

**Figure 1:**
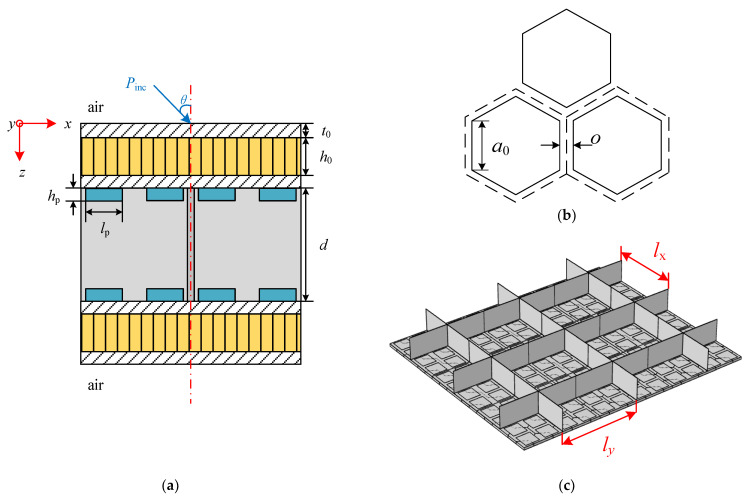
Schematic diagram of an orthogonally rib-stiffened honeycomb double sandwich structure with periodic arrays of shunted piezoelectric patches: (**a**) Side view of a periodic element of the research structure; (**b**) Honeycomb structure; (**c**) Orthogonal rib-stiffeners.

Figure 1 presents a schematic diagram of the research model, which is an infinite two-dimensional sandwich structure. Figure 1a is the side view of a periodic element of the research structure. It can be seen from the figure that the structure consists of two parallel honeycomb sandwich structures pasted with periodic arrays of shunted piezoelectric patches on the inner side, and the two parallel honeycomb sandwich structures are reinforced by an orthogonally rib-stiffened core which has two periodic uniform spacings of *l_x_* and *l_y_* in the *x*- and *y*-directions, respectively. The geometric parameters of the research structure are the wall thickness of the honeycomb core cell *o*, side length of the honeycomb core cell *a_*0*_*, thickness of facing skin *t_*0*_* (assuming that both skins are of same each other), height of honeycomb core *h_*0*_*, thickness of *x*- and *y*-wise stiffeners *t_x_* and *t_y_*, height of orthogonal rib-stiffeners *d*, length and width of piezoelectric patches *l_p_*, and thickness of piezoelectric patches *h_p_*. 

The two parallel honeycomb sandwich structures are placed in an infinite air space. A right-handed Cartesian coordinate system (*x*, *y*, *z*) is established on the upper surface of the top honeycomb sandwich plate. The positive directions of the *x*-axis and *y*-axis are along the two directions of the orthogonal stiffeners respectively, and the positive direction of the *z*-axis is vertical downward.

A plane sound wave *P_inc_* varying harmonically in time is incident upon the top honeycomb sandwich plate of the structure with azimuth angle *θ* and elevation angle *φ*, inducing a bending wave that propagates along the plate. The bending wave can be transmitted from the top plate to the bottom plate through two paths: the airborne path through the middle sound field and the structure-borne through the orthogonal stiffeners. In addition, the tensile, torsional, and bending motions of the stiffeners and their corresponding inertial effects are also considered in the proposed model.

### 2.2. Equivalent Characteristics of the Structure

**Figure 2:**
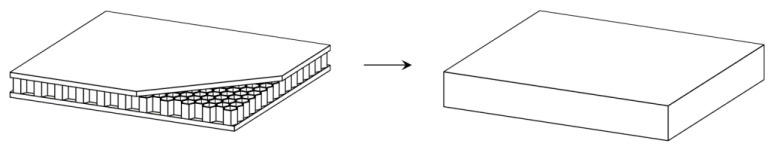
Schematic diagram of the equivalent transformation.

Honeycomb sandwich structures have been widely used in the fields of spaceflight and aviation because of their light weight and excellent sound insulation performance. Due to the complexity of such structures, a certain degree of simplification and equivalence are often required in the analysis. In this work, the honeycomb sandwich structures are equivalent using Hoff theory. According to Hoff theory, the honeycomb sandwich plate is equivalent to an isotropic shell with different thickness from the original sandwich plate, which is shown in Figure 2. The formulas of equivalent mechanical parameters are as follows [29,30]:

(1) $$\upsilon_{eq}=\upsilon_{f}$$

(2) $$H_{eq}=\sqrt{t_{0}^{2}+3{\left(h_{0}+t_{0}\right)}^{2}}$$

(3) $$E_{eq}=\frac{2E_{f}t_{0}}{H_{eq}}$$

(4) $$\rho_{ceq}=\frac{2}{\sqrt{3}}\frac{o}{a_{0}}\rho_{c}$$

(5) $$\rho_{eq}=\frac{k(2t_{0}\rho_{f}+h_{0}\rho_{ceq})}{H_{eq}}$$

where *E_f_*, *υ_f_*, and *ρ_f_* are the Young’s modulus, Poisson ratio, and density of the facing skin of honeycomb sandwich structures, respectively. *E_eq_*, *υ_eq_*, *ρ_eq_*, and *H_eq_* are the Young’s modulus, Poisson ratio, density, and height of the equivalent plate, *ρ_ceq_* is density of the honeycomb core after equivalence, *ρ_c_* is the density of the material of honeycomb core, and *k* is a modified parameter, which is 1.5.

**Figure 3:**
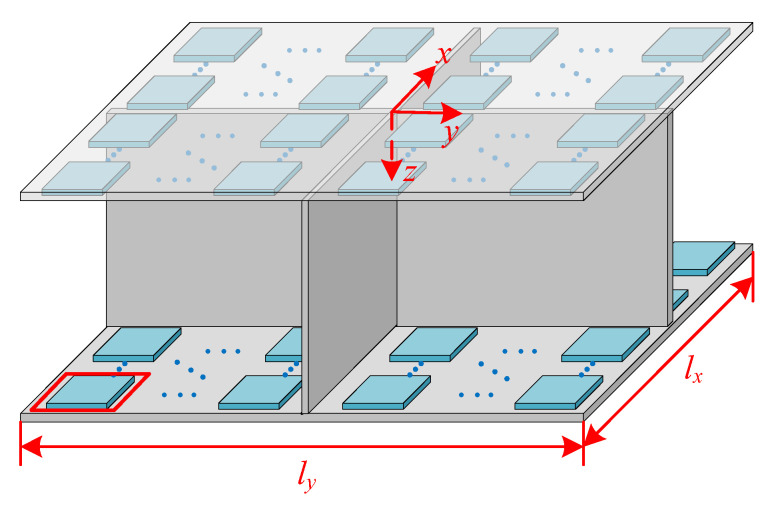
Detail view of a periodic element of the research structure (the top plate is transparent).

**Figure 4:**
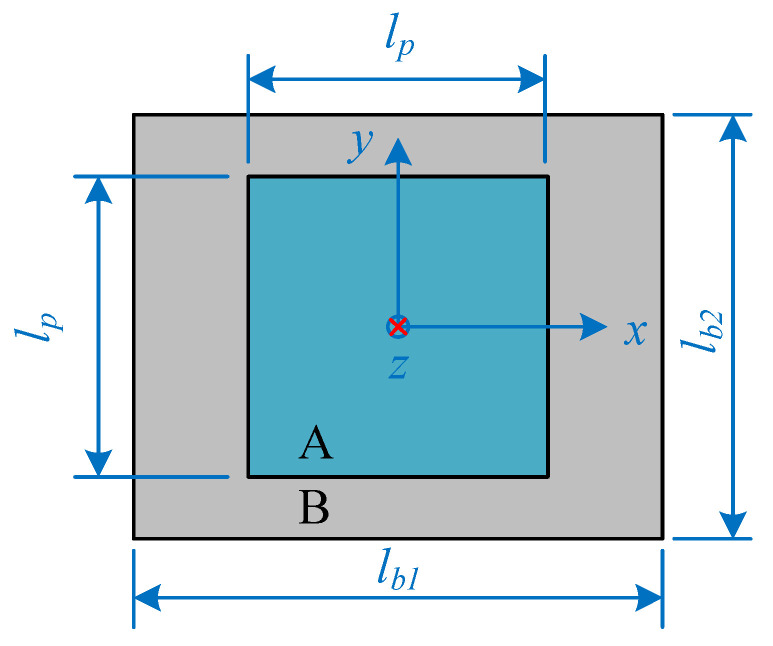
Top view of the basic unit of periodic shunted piezoelectric patches structure.

The research structure shown in Figure 1 has spatial periodicity, so only the periodic elements shown in Figure 3 need to be studied. The upper and lower plates have the same structure layouts, and the inner side of each plate contains *a* rows and *a* columns of periodic shunted piezoelectric patch arrays which are composed of *a*^2^ pieces of piezoelectric patches. The basic unit of the periodic shunted piezoelectric patch structure is shown in the red box in Figure 3, and the top view of the basic unit is shown in Figure 4. The basic unit is divided into two regions. The region pasted with shunted piezoelectric patch is denoted by A, while the single layer region is denoted by B. The length and width of the substrate are *l_b*1*_* and *l_b*2*_*, respectively, and the side length of the square shunted piezoelectric patch is *l_p_*.

In order to analyze the sound insulation characteristics of the structure, the equivalent physical parameters of the whole structure must be calculated by the EM method first. The calculation process of equivalent parameters can be divided into the following steps.

First, the effective material parameters of a single piezoelectric patch with external shunt circuit need to be determined. The piezoelectric patch can be treated as an isotropic plate, and the Young’s modulus and Poisson ratio of it are *E_p,i_* and *υ_p,i_* respectively. Their formulas are as follows [31]:

(6) $$\begin{array}{l} E_{p,i}=\frac{h_{p,i}(1+sZ_{i}C_{p,i})}{h_{p,i}s_{11,i}^{E}(1+sZ_{i}C_{p,i})-sZ_{i}d_{31,i}^{2}A_{p,i}}\\ \upsilon_{p,i}=-\frac{s_{12,i}^{E}(1+sZ_{i}C_{p,i})-sZ_{i}d_{31,i}^{2}A_{p,i}h_{p,i}^{-1}}{s_{11,i}^{E}(1+sZ_{i}C_{p,i})-sZ_{i}d_{31,i}^{2}A_{p,i}h_{p.i}^{-1}} \end{array}$$

where *i* represents the *i*th piezoelectric patch (*i* = 1, 2, …, *a*^2^), *ω* = 2*πf* is the angular frequency and *s* = *jω* is the Laplace operator (*j* = $\sqrt{-1}$). *A_p,i_* and *h_p,i_* denote the area and thickness of the *i*th shunted piezoelectric patch respectively. *d*_31*,i*_ and $s_{11,i}^{E}$ are the *i*th piezoelectric constant and piezoelectric patch compliance coefficient respectively, *Z_i_* is the impedance of the shunting circuit connected to the piezoelectric patch. The three components of the corresponding parameter are denoted by subscripts 1, 2, and 3. The direction of vector is along the three coordinate axes. The inherent capacitance of *i*th piezoelectric patch under constant stress is denoted by *C_p,i_*, which can be expressed as:

(7) $$C_{p,i}=\frac{A_{p,i}\varepsilon_{33,i}^{T}}{h_{p,i}}$$

where $\varepsilon_{33,i}^{T}$ is the dielectric constant of the *i*th piezoelectric patch under constant stress.

Second, the equivalent surface density and equivalent dynamic bending stiffness of regions *A* and *B* of the unit cell shown in Figure 4 are given by:

(8) $$\begin{array}{l} \sigma_{j}(x,y)=\left\{ \begin{array}{l} \sigma_{A,j}=\rho_{eq,j}H_{eq,j}+\rho_{p,j}h_{p,j},\;(x,y)\in A\\ \sigma_{B,j}=\rho_{eq,j}H_{eq,j},\;(x,y)\in B \end{array}\right.\\ D_{j}(x,y)=\left\{ \begin{array}{l} D_{A,j}=\frac{E_{eq,j}H_{eq,j}^{3}}{12(1-\upsilon_{eq,j}^{2})}+\frac{E_{p,i}\left[{\left(H_{eq,j}+2h_{p,i}\right)}^{3}-H_{eq,j}^{3}\right]}{24(1-\upsilon_{p,j}^{2})},\;(x,y)\in A\\ D_{B,j}=\frac{E_{eq,j}H_{eq,j}^{3}}{12(1-\upsilon_{eq,j}^{2})},\;(x,y)\in B \end{array}\right. \end{array}$$

where *ρ_p,j_* is density of the shunted piezoelectric patch, while *j* = 1 and 2 represent the upper and lower periodic plate.

Third, according to the EM method, the equivalent surface mass density and equivalent dynamic bending stiffness of the periodic plate can be written as [19]:

(9) $$\begin{array}{l} \sigma_{eq,j}=\alpha \sigma_{A,j}+(1-\alpha )\sigma_{B,j}\\ D_{eq,j}=\frac{D_{A,j}D_{B,j}}{(1-\alpha )D_{A,j}+\alpha D_{B,j}} \end{array}$$

where *α* represents the ratio between the total area of region *A* and the total area of the basic unit, given by:

(10) $$\alpha =\frac{l_{p}{}^{2}}{l_{b1}l_{b2}}$$

### 2.3. Analysis of Sound Transmission and Panel Vibration

Under the excitation of a harmonic plane sound wave, the responses of the upper and lower plates of the sandwich structure can be expressed by a spatial harmonic expansion, as follows [5,8,31,32]:

(11) $$w_{j}(x,y,t)=\sum_{m=-\infty }^{+\infty }\sum_{n=-\infty }^{+\infty }\alpha_{j,mn}e^{-i[(k_{x}+2m\pi /l_{x})x+(k_{y}+2n\pi /l_{y})y-\omega t]}$$

where subscripts *j* = 1 and 2 denote the upper and lower periodic plates. The (*m*, *n*)th harmonic wave components in the two plates have the same wavenumbers (*k_x_ +* 2*mπ*/*l_x_*, *k_y_ +* 2*nπ*/*l_y_*) but different amplitudes:

(12) $$\alpha_{j,mn}=\frac{1}{l_{x}l_{y}}\int_{0}^{l_{x}}\int_{0}^{l_{y}}w_{j}(x,y,t)e^{i[(k_{x}+2m\pi /l_{x})x+(k_{y}+2n\pi /l_{y})y-\omega t]}dxdy$$

The wavenumbers *k_x_ +* 2*mπ*/*l_x_* > 0, *k_y_ +* 2*nπ*/*l_y_* > 0 stand for positive-going harmonic waves in the x- and y-direction and vice versa. The (*m*, *n*)th space harmonic wavenumber in the z-direction is denoted by *k_z,mn_*, which is given by [33,34]:

(13) $$k_{z,mn}=\sqrt{{\left(\frac{\omega }{c_{0}}\right)}^{2}-{\left(k_{x}+\frac{2m\pi }{l_{x}}\right)}^{2}-{\left(k_{y}+\frac{2n\pi }{l_{y}}\right)}^{2}}$$

The pressure waves become evanescent waves when (*ω*/*c*_0_)^2^ < (*k_x_* + 2*mπ*/*l_x_*)^2^ + (*k_y_* + 2*nπ*/*l_y_*)^2^, and then the expression is replaced by:

(14) $$k_{z,mn}=i\sqrt{{\left(k_{x}+\frac{2m\pi }{l_{x}}\right)}^{2}+{\left(k_{y}+\frac{2n\pi }{l_{y}}\right)}^{2}-{\left(\frac{\omega }{c_{0}}\right)}^{2}}$$

### 2.4. Motion Analysis of Periodic Panels and Stiffeners

**Figure 5:**
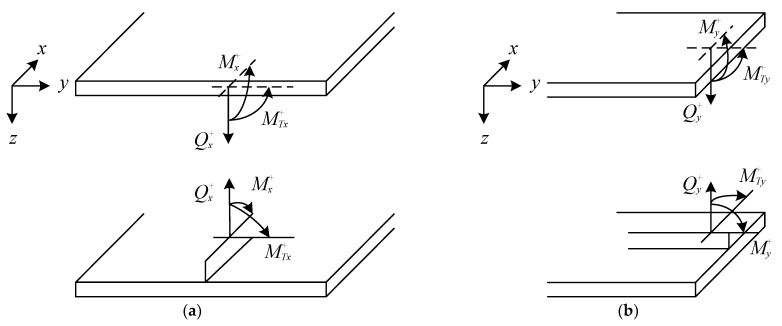
Conventions for torsional moments, bending moments and tensional forces between the upper panel and (**a**) *x*-wise; (**b**) *y*-wise rib-stiffeners.

There is a strong constraint imposed by the orthogonal rib-stiffeners on the motions of the plates. Thus, to accurately simulate the acoustic behavior and vibration of the research structure, the influence of torsional moments, bending moments, and tensional forces imposed on the connected plates should be taken into account. To represent these forces and moments, as can be seen from Figure 5, ($Q_{x}^{+}$, $M_{x}^{+}$, $M_{Tx}^{+}$) and ($Q_{y}^{+}$, $M_{y}^{+}$, $M_{Ty}^{+}$) are applied to denote the impacts at the interface between the upper plate and *x*/*y*-wise stiffeners. Moreover, ($Q_{x}^{-}$, $M_{x}^{-}$, $M_{Tx}^{-}$) and ($Q_{y}^{-}$, $M_{y}^{-}$, $M_{Ty}^{-}$) denote the loads at the interfaces between the lower plate and *x*/*y*-wise stiffeners, which is similar to the interface between the upper plate and is not shown for brevity. For detailed formulas of these forces and moments, the reader can refer to the literature [8,31,35].

### 2.5. Application of Virtual Work Principle in Solving the Formulations

Based on one periodic element, coefficients *α*_1*,mn*_ and *α*_2*,mn*_ can be determined by applying the virtual work principle [5,8,33,36]. According to the virtual work principle, the expressions of virtual placements of the upper and lower plates are given by:

(15) $$\delta w_{j}=\delta \alpha_{j,mn}e^{-i[(k_{x}+2m\pi /l_{x})x+(k_{y}+2n\pi /l_{y})y]}\;(j=1,2)$$

where *δ* is the Dirac delta function.

In one period of the structure, the equations governing the vibration responses of the periodic plates are:

(16) $$\begin{array}{l} D_{eq,1}\nabla^{4}w_{1}+\sigma_{eq,1}\frac{\partial^{2}w_{1}}{\partial t^{2}}-P_{1}(x,y,0)+P_{2}(x,y,h_{1})=0\\ D_{eq,2}\nabla^{4}w_{2}+\sigma_{eq,2}\frac{\partial^{2}w_{2}}{\partial t^{2}}-P_{2}(x,y,h_{1}+d)+P_{3}(x,y,h_{1}+d+h_{2})=0 \end{array}$$

where *P*_1_(*x*, *y*, 0), *P*_2_(*x*, *y*, *h*_1_), *P*_2_(*x*, *y*, *h*_1_ + *d*), and *P*_3_(*x*, *y*, *h*_1_ + *d* + *h*_2_) are the sound pressure in the top, middle, and bottom sound field, respectively. For detailed formulas of these sound pressure, the reader can refer to the literature [5,8,31].

The virtual work principle requires that

(17) $$\delta \prod_{pr}+\delta \prod_{xr}+\delta \prod_{yr}=0,\;(r=1,2)$$

where subscripts *pr*, *xr,* and *yr* represent the virtual works of the panel elements, the *x*-wise rib-stiffeners, and the *y*-wise rib-stiffeners, respectively. For detailed formulas of these virtual works, the reader can refer to the literature [8,31].

It can be obtained by solving (17) that

(18) $$\begin{array}{l} \left[D_{eq,1}{\left(\alpha_{k}^{2}+\beta_{l}^{2}\right)}^{2}-\sigma_{eq,1}\omega^{2}-\frac{\omega^{2}\rho_{0}}{ik_{z,kl}}+\frac{\omega^{2}\rho_{0}\cos(k_{z,kl}d)}{k_{z,kl}\sin(k_{z,kl}d)}\right]l_{x}l_{y}\alpha_{1,kl}-\frac{\omega^{2}\rho_{0}}{k_{z,kl}\sin(k_{z,kl}d)}l_{x}l_{y}\alpha_{2,kl}\\ +\sum_{n=-\infty }^{+\infty }\left[R_{Q1}-i\alpha_{k}^{3}R_{M1}-i\beta_{l}\alpha_{k}\beta_{n}R_{T1}\right]l_{x}\alpha_{1,kn}+\sum_{n=-\infty }^{+\infty }\left[-R_{Q2}+i\alpha_{k}^{3}R_{M2}+i\beta_{l}\alpha_{k}\beta_{n}R_{T2}\right]l_{x}\alpha_{2,kn}\\ +\sum_{m=-\infty }^{+\infty }\left[R_{Q3}-i\beta_{l}^{3}R_{M3}-i\alpha_{k}\alpha_{m}\beta_{l}R_{T3}\right]l_{y}\alpha_{1,ml}+\sum_{m=-\infty }^{+\infty }\left[-R_{Q4}+i\beta_{l}^{3}R_{M4}+i\alpha_{k}\alpha_{m}\beta_{l}R_{T4}\right]l_{y}\alpha_{2,ml}\\ =\left\{ \begin{array}{l} 2Il_{x}l_{y}\;\mathrm{when}\;k=0\&l=0\\ 0\;\mathrm{when}\;k\neq 0∥l\neq 0 \end{array}\right. \end{array}$$

(19) $$\begin{array}{l} \left[D_{eq,2}{\left(\alpha_{k}^{2}+\beta_{l}^{2}\right)}^{2}-\sigma_{eq,2}\omega^{2}-\frac{\omega^{2}\rho_{0}}{ik_{z,kl}}+\frac{\omega^{2}\rho_{0}\cos(k_{z,kl}d)}{k_{z,kl}\sin(k_{z,kl}d)}\right]l_{x}l_{y}\alpha_{2,kl}-\frac{\omega^{2}\rho_{0}}{k_{z,kl}\sin(k_{z,kl}d)}l_{x}l_{y}\alpha_{1,kl}\\ +\sum_{n=-\infty }^{+\infty }\left[-R_{Q2}+i\alpha_{k}^{3}R_{M2}+i\beta_{l}\alpha_{k}\beta_{n}R_{T2}\right]l_{x}\alpha_{1,kn}+\sum_{n=-\infty }^{+\infty }\left[R_{Q1}-i\alpha_{k}^{3}R_{M1}-i\beta_{l}\alpha_{k}\beta_{n}R_{T1}\right]l_{x}\alpha_{2,kn}\\ +\sum_{m=-\infty }^{+\infty }\left[-R_{Q4}+i\beta_{l}^{3}R_{M4}+i\alpha_{k}\alpha_{m}\beta_{l}R_{T4}\right]l_{y}\alpha_{1,ml}+\sum_{m=-\infty }^{+\infty }\left[R_{Q3}-i\beta_{l}^{3}R_{M3}-i\alpha_{k}\alpha_{m}\beta_{l}R_{T3}\right]l_{y}\alpha_{2,ml}=0 \end{array}$$

where *R_T_*_1_, *R_T_*_2_, *R_T_*_3_, and *R_T_*_4_ are torsional moment coefficients, *R_M_*_1_, *R_M_*_2_, *R_M_*_3_, and *R_M_*_4_ are bending moment coefficients, and *R_Q_*_1_, *R_Q_*_2_, *R_Q_*_3_, and *R_Q_*_4_ are tensional force coefficients. For a detailed derivation of these parameters, the reader can refer to the literature [8,31]. The expressions of *α_m_* and *β_n_* are as follows:

(20) $$\begin{array}{l} \alpha_{m}=k_{x}+\frac{2m\pi }{l_{x}}\\ \beta_{n}=k_{y}+\frac{2n\pi }{l_{y}} \end{array}$$

Formulas (18) and (19) form an infinite set of coupled equations. In order to obtain the solution, the equations need to be truncated, namely the sum-index (*m*, *n*) should be limited in the finite ranges of *m* = $-\hat{k}\sim \hat{k}$ and *n* = $-\hat{l}\sim \hat{l}$. Thus, the infinite coupled equations can be grouped into a finite order matrix form (i.e., 2*KL*, where *K* = $2\hat{k}+1$, *L* = $2\hat{l}+1$) as shown below:

(21) $${\left[\begin{array}{cc} T_{11} & T_{12}\\ T_{21} & T_{22} \end{array}\right]}_{2KL\times 2KL}{\left\{\begin{array}{c} \alpha_{1,kl}\\ \alpha_{2,kl} \end{array}\right\}}_{2KL\times 1}={\left\{\begin{array}{c} F_{kl}\\ 0 \end{array}\right\}}_{2KL\times 1}$$

For the detailed derivation of (21), refer to the literature [8,33,35,36].

### 2.6. Sound Transmission Loss

The sound transmission coefficient can be expressed as follows:

(22) τ(φ,θ)=∑m=−∞+∞∑n=−∞+∞|ξmn|2Re(kz,mn)|I|2kz

which is dependent on the elevation angle *φ* and azimuth angle *θ*, where *I* denotes the amplitude of incident sound pressure, Re(*k_z,mn_*) denotes the real part of *k_z,mn_*, and *ξ_mn_* is a relevant coefficient, which is given by:

(23) $$\xi_{mn}=-\frac{\omega^{2}\rho_{0}\alpha_{2,mn}}{ik_{z,mn}}e^{ik_{z,mn}(h_{1}+h_{2}+d)}$$

The sound transmission loss (STL) may be defined as the inverse of the power transmission coefficient in decibels scale [37].

(24) $$STL=10\log_{10}\left(\frac{1}{\tau (\varphi ,\theta )}\right)$$

## 3. Parametric Investigation and Discussions

### 3.1. Basic Parameters and Design of External Circuit

**Figure 6:**
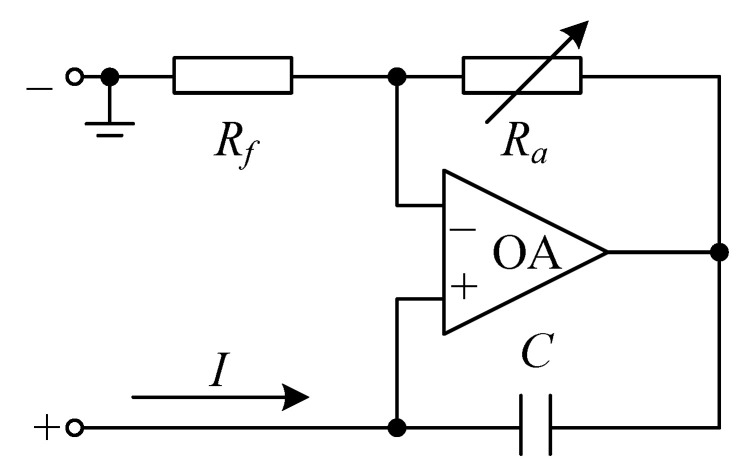
Equivalent negative capacitance circuit.

For the structure shown in Figure 1, 16 pieces of piezoelectric patches (i.e., *a* = 4) are pasted on the inner sides of each plate, and it is assumed that they are connected with the identical shunting circuit shown in Figure 6, respectively. The circuit is an equivalent negative capacitance shunting circuit, which is composed of an adjustable resistor (*R_a_*), a fixed resistor (*R_f_*), an operational amplifier (OA), and a capacitor (C), where *R_f_* consists of the external shunt resistance and internal resistance of the PZT (piezoelectric transducer) patch. The equivalent negative capacitance can be written as [31]

(25) $$-C_{eq}=-\frac{R_{f}}{R_{a}}C$$

Thus, the impedance of each shunting circuit is given by

(26) $$Z_{i}=\frac{1}{-C_{eq}\cdot s}$$

where *s* = *jω* is the Laplace operator (*j* =$\sqrt{-1}$). In the research structure, the type of piezoelectric patch is PZT_5H, and aluminum is selected as the material of the stiffeners and honeycomb sandwich structures. The model physical parameters of the PZT patch and the honeycomb sandwich structure are listed in Table 1.

### 3.2. Conditions for Model Application

The prerequisite for the establishment of the EM method is to meet the subwavelength hypothesis [19,31]. It is known that the unit cell shown in Figure 4 meets the subwavelength hypothesis through verification.

In order to make the solution of infinite governing Equation (21) converge, sufficient numbers of *m* and *n* need to be used. The description of the admissible criterion is when the solution converges at a given frequency. It is convergent for all frequencies below that frequency. Therefore, it is only necessary to determine the number of the truncations at the highest frequency (i.e., 2000 Hz). Thus, the convergence check is carried out by calculating STL at 2000 Hz, and then adding more terms to further calculate the corresponding STL until the difference between two consecutive calculated values falls within the preset error range (0.01 dB in this work), the solution can be considered convergent. Furthermore, at all other frequencies below 2000 Hz, the corresponding number of terms is also suitable to calculate STL.

**Figure 7:**
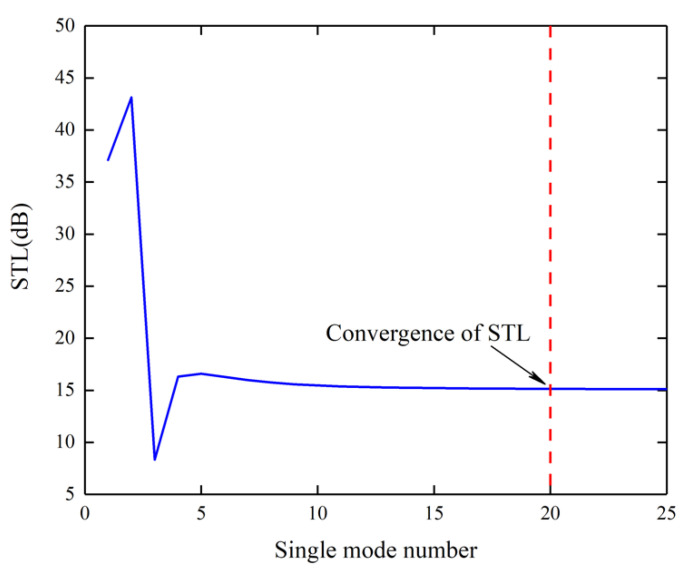
Convergence check of the research structure at 2000 Hz.

The equations can be simplified to a finite size with *m* = $-\hat{k}\sim \hat{k}$ and *n* = $-\hat{l}\sim \hat{l}$ ($\hat{k}=\hat{l}$ assumed) because of the symmetry of the research structure in x- and y-directions. As can be seen from Figure 7, with the increase of the number of single modes, the solution of STL at 2000 Hz gradually tends to converge. Therefore, when $\hat{k}\geq$ 20, at least 1681 terms are required to make the solution converge at 2000 Hz.

### 3.3. Model Validation

**Figure 8:**
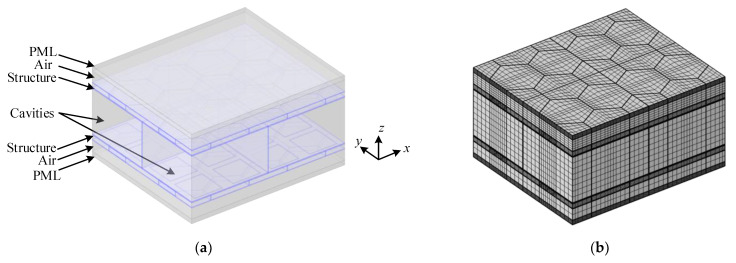
FE model of one periodic element without piezoelectric patches: (**a**) transparent model and (**b**) mesh model.

To demonstrate the validation of model, the theoretical values are compared with the COMSOL Multiphysics 5.4 finite element simulation (FE) results. The acoustic-structure coupling FE model of one periodic element in the model without PZT patches is established, as shown in Figure 8. The research structure shown in blue is arranged in the air flow field and the air cavities divided by the orthogonal rib-stiffeners in the middle sound field contain the same medium as the external sound fields, in which all the interfaces are simulated by the acoustic-structure boundaries. The perfectly matched layer (PML) is used to approximate the two semi-infinite sound fields at the upper and lower of the model respectively. In the *x*- and *y*-directions of the model, Floquet periodicity boundaries are applied to approach the infinite sandwich structure. The structure is divided using swept meshes.

**Figure 9:**
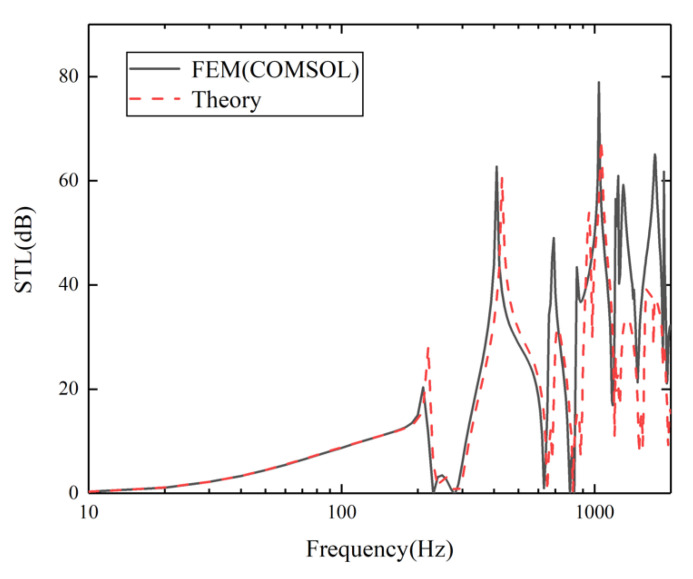
Comparison of the theoretical values and simulation results for the research model without piezoelectric patches.

Figure 9 shows that there is a similar trend between the curve of theoretical calculation and the curve of finite element simulation. Although there are visible discrepancies between the simulation curve and the theoretical curve in the high frequency part, the corresponding valley frequencies (such as those around 235.2 Hz, 645.4 Hz, and 825.3 Hz) and peak frequencies (such as those around 215.1 Hz, 425.3 Hz, and 715.4 Hz) can match well. With the increase of frequency, the calculation accuracy of the EM model decreases, resulting in visible discrepancies at relatively high frequencies. Overall, the discrepancies between the two curves is within an acceptable range. Therefore, the correctness of the theoretical model can be demonstrated.

### 3.4. Validity of the Research Structure

In order to verify the validity of the research structure, the orthogonal stiffened sandwich structure with the same material parameters and geometric characteristics but without piezoelectric patches is used as the comparison model. The parameters are shown in Table 1.

**Figure 10:**
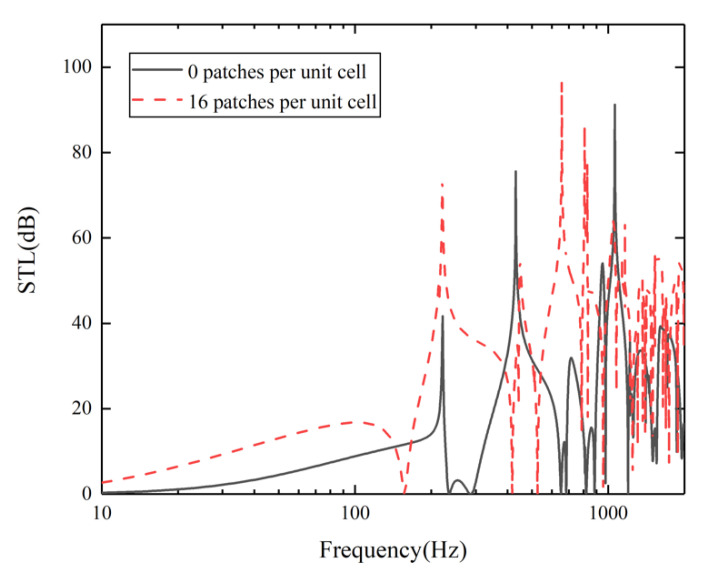
Comparison of the STL curves between the research model with 16 piezoelectric patches per unit cell and the comparison model without piezoelectric patches.

A comparison of STL curves of the two models at 10–2000 Hz is shown in Figure 10. It can be seen from the figure that the sound insulation of the research structure in the whole frequency band is better than that of the comparison model without piezoelectric patches. In addition, the first valley frequency is moved from 236.5 Hz to 157.1 Hz, which makes the sound insulation effect of the research structure in the low frequency range significantly better than that of the structure without piezoelectric patches. This is because piezoelectric patches transform the energy of noise and vibration into electrical energy, which is then consumed by shunt circuits, thus improving the STL [19]. Therefore, by introducing piezoelectric patches, the STL in low-frequency is improved and the sound insulation bandwidth of the structure is broadened.

## 4. Influence Law of Parameters

In the previous section, the correctness of the research model is verified. On this basis, the factors affecting STL are further studied.

### 4.1. Influence of Incident Angles

**Figure 11:**
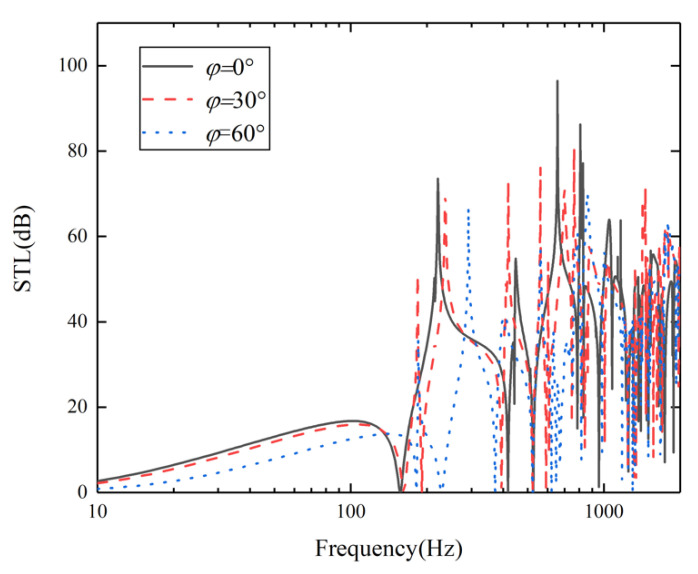
STL at different incident angles.

According to the research structure, the effect of incident azimuth is negligible here. Therefore, when the azimuth angle is constant at 0°, the sound insulation curves with incident angles of 0°, 30°, and 60° are studied in the range of 10–2000 Hz, and the results are shown in Figure 11. The figure indicates that with the increase of incident angle, the STL in the whole frequency band decreases gradually. This is because the waveform changes when the sound wave incidents on the panel obliquely, resulting in the change of space harmonic wavenumber in *z*-direction [31]. Finally, with the increase of acoustic incident angle, the acoustic transmission coefficient increases. It can also be seen from Figure 11 that the first valley frequency tends to move gradually to a higher frequency.

### 4.2. Influence of the Height of Honeycomb Core

**Figure 12:**
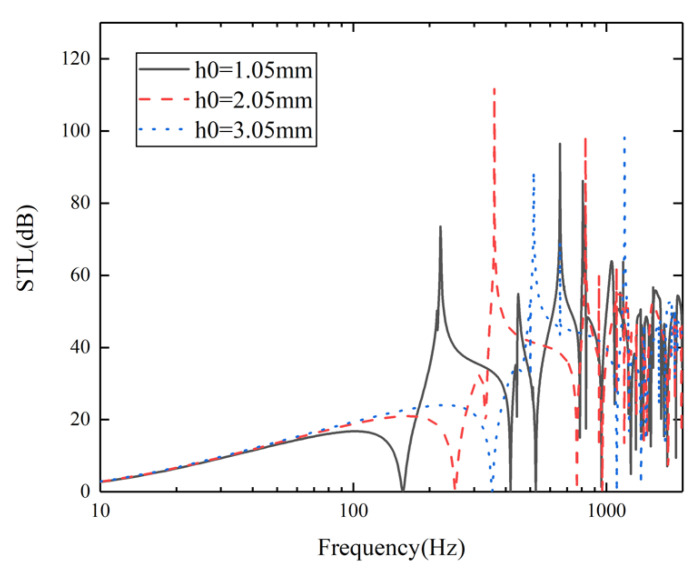
STL at different height of honeycomb core.

To explore the influence of the height of the honeycomb core on sound insulation, three cases of *h*_0_ = 1.05 mm, 2.05 mm, and 3.05 mm are selected on the premise that the honeycomb core height of the upper and lower sandwich plates is equal. The STL curves of the three cases are shown in Figure 12. The figure shows that when *h*_0_ is 1.05 mm, 2.05 mm, and 3.05 mm, respectively, the corresponding first valley frequency is 157.1 Hz, 253.1 Hz, and 253.3 Hz. It can be seen that with the increase of honeycomb core height *h*_0_, the first valley frequency tends to move to a higher frequency. This is because the equivalent dynamic bending stiffness and equivalent surface mass density of the equivalent plate change as the height of honeycomb core increases [31]. Moreover, the movement of the first valley frequency also leads to the gradual narrowing of the sound insulation bandwidth.

## 5. Conclusions

This paper is concerned with the sound transmission loss (STL) of a new composite structure, which is an orthogonally rib-stiffened honeycomb double sandwich structure with periodic arrays of shunted piezoelectric patches. To explore the acoustic characteristics of the structure, the research model needs to be equivalent according to Hoff theory and the EM method. Following the two theories, the sandwich panels are treated as homogeneous plates with equivalent surface density and equivalent dynamic bending stiffness. Thus, the STL can be predicted efficiently. From the comparison of the curve of theoretical calculation and the curve of finite element simulation, it can be found that the corresponding valley frequencies and peak frequencies of the two curves can effectively match, which can prove the correctness of the theoretical model. By comparing the STL curves of the complete model and the comparison model without the piezoelectric patches, it can be found that the research sandwich structure demonstrates a better sound insulation effect at low frequencies. In addition, several conclusions are obtained through the study of the influences of sound incident angles and height of honeycomb core on the research structure. First, with the increase of incident angle, the STL in the whole frequency band decreases gradually, and the first valley frequency has a tendency to move gradually to a higher frequency. Second, as the height of the honeycomb core increases, the first valley frequency tends to move to a higher frequency, and the sound insulation bandwidth gradually narrows.

## Figures and Tables

**Table 1:** Properties of one periodic element.

Parameter	Value	Parameter	Value
o (mm)	1	*t_x_* & *t_y_* (mm)	1
*a*_0_ (mm)	40	*l*_b1_ (mm)	*l_x_*/4
*t*_0_ (mm)	1	*l*_b2_ (mm)	*l_y_*/4
*h*_0_ (mm)	0.1	*ρ_p_* (kg/m^3^)	7500
*l_x_* (mm)	$4\sqrt{3}a_{0}+4o$	*l_p_* (mm)	50
*l_y_* (mm)	$6a_{0}+2\sqrt{3}o$	*h_p_* (mm)	1
*d* (mm)	80	$s_{E\cdot 11}$/(m^2^/N)	16.5 × 10^−12^
$s_{E\cdot 12}$/(m^2^/N)	−4.78 × 10^−12^	*E_x_* & *E_y_* (Gpa)	70
*d*_31_/(C/N)	−2.74 × 10^−10^	*φ* (°)	0
$\varepsilon_{33}^{T}$/(F/m)	3.01 × 10^−8^	*θ* (°)	0
−*C*_eq_ (F)	−0.3*C_p_*	*E_f_* (Gpa)	70
*G_x_* & *G_y_* (Gpa)	27		
*ρ_x_* & *ρ_y_* (kg/m^3^)	2700		

## Data Availability

The data used to support the findings of this study are included within the article.

## References

[1] Donaldson B.K. (1973). A new approach to the forced vibration of flat skin-stringer-frame structures. J. Sound Vib..

[2] Cao X., Hua H. (2016). Acoustic responses of the composite sandwich plates with lattice truss core to the subsonic turbulent boundary layer. Compos. Struct..

[3] Mead D.M. (1996). Wave propagation in continuous periodic structures: Research contributions from Southampton, 1964–1995. J. Sound Vib..

[4] Dozio L., Ricciardi M. (2009). Free vibration analysis of ribbed plates by a combined analytical–numerical method. J. Sound Vib..

[5] Mead D.J., Pujara K.K. (1971). Space-harmonic analysis of periodically supported beams: Response to convected random loading. J. Sound Vib..

[6] Mace B.R. (1980). Periodically stiffened fluid-loaded plates I: Response to convected harmonic pressure and free wave propagation. J. Sound Vib..

[7] Mead D.J. (1970). Free wave propagation in periodically supported, infinite beams. J. Sound Vib..

[8] Xin F.X., Lu T.J. (2010). Analytical modeling of fluid loaded orthogonally rib-stiffened sandwich structures: Sound transmission. J. Mech. Phys. Solids.

[9] Kelsey S., Gellatly R.A., Clark B.W. (1958). The shear modulus of foil honeycomb cores. Aircr. Eng. Aerosp. Technol..

[10] Kobayashi H., Daimaruya M., Okuto K. (1994). Elasto-plastic bending deformation of welded honeycomb sandwich panel. Trans. Jpn. Soc. Mech. Eng..

[11] Kunimoto T., Yamada H. (1987). Study on the buffer characteristics of the honeycomb sandwich construction under dynamic loading. J. Jpn. Inst. Light Met..

[12] Kunimoto T., Mori N. (1989). Study on the buffer characteristics of the corrugated-core used for the 5052 aluminum alloy sandwich construction under dynamic loading. J. Jpn. Inst. Light Met..

[13] Forward R.L. (1979). Electronic damping of vibrations in optical structures. Appl. Opt..

[14] Hagood N.W., Flotow A.V. (1991). Damping of structural vibrations with piezoelectric materials and passive electrical networks. J. Sound Vib..

[15] Ahmadian M., Jeric K.M. (2001). On the application of shunted piezo ceramics for increasing acoustic transmission loss in structures. J. Sound Vib..

[16] Kim J., Lee J. (2002). Broadband transmission noise reduction of smart panels featuring piezoelectric shunt circuits and sound-absorbing material. J. Acoust. Soc. Am..

[17] Kim J., Choi J.Y. (2005). Performance test for transmitted noise reduction of smart panels using piezoelectric shunt damping. Smart Mater. Struct..

[18] Kim J., Jung Y.C. (2006). Broadband noise reduction of piezoelectric smart panel featuring negative-capacitive-converter shunt circuit. J. Acoust. Soc. Am..

[19] Zhang H., Wen J., Xiao Y., Wang G., Wen X. (2015). Sound transmission loss of metamaterial thin plates with periodic subwavelength arrays of shunted piezoelectric patches. J. Sound Vib..

[20] Casadei F., Dozio L., Ruzzene M., Cunefare K.A. (2010). Periodic shunted arrays for the control of noise radiation in an enclosure. J. Sound Vib..

[21] Wu S.Y. (1998). Method for multiple mode piezoelectric shunting with single PZT transducer for vibration control. J. Intell. Mater. Syst. Struct..

[22] Behrens S., Moheimani S.O.R., Fleming A.J. (2003). Multiple mode current flowing passive piezoelectric shunt controller. J. Sound Vib..

[23] Berardengo M., Manzoni S., Conti A.M. (2017). Multi-mode passive piezoelectric shunt damping by means of matrix inequalities. J. Sound Vib..

[24] Moheimani S.O.R., Fleming A.J., Behrens S. (2004). Dynamics, stability, and control of multivariable piezoelectric shunts. IEEE/ASME Trans. Mechatron..

[25] Fleming A.J., Moheimani S.O.R. (2004). Control orientated synthesis of high-performance piezoelectric shunt impedances for structural vibration control. IEEE Trans. Control. Syst. Technol..

[26] Bricault C., Pézerat C., Collet M., Pyskir A., Perrard P., Matten G., Romero-García V. (2019). Multimodal reduction of acoustic radiation of thin plates by using a single piezoelectric patch with a negative capacitance shunt. Appl. Acoust..

[27] Anish Kumar A., Chakrabarti A. (2018). Influence of openings and additional mass on vibration of laminated sandwich rhombic plates using IHSDT. J. Thermoplast. Compos. Mater..

[28] Talebitooti R., Zarastvand M.R. (2018). Vibroacoustic behavior of orthotropic aerospace composite structure in the subsonic flow considering the third order shear deformation theory. Aerosp. Sci. Technol..

[29] Cheng Y., Zeng Q., Liu H., Fang J., Rao C. (2014). Optimized analysis of geometry parameters for honeycomb sandwich mirror Advances in Optical and Mechanical Technologies for Telescopes and Instrumentation. Int. Soc. Opt. Photonics.

[30] Xian-Bing L.I., Wen J.H., Dian-Long Y.U., Wen X.S. (2012). The comparative study of equivalent mechanical methods on honeycomb sandwich plate. Fiber Reinf. Plast.Compos..

[31] Zhifu Z., Weiguang Z., Qibai H. (2017). Low-frequency broadband sound transmission loss of infinite orthogonally rib-stiffened sandwich structure with periodic subwavelength arrays of shunted piezoelectric patches. Shock. Vib..

[32] Mace B.R. (1981). Sound radiation from fluid loaded orthogonally stiffened plates. J. Sound Vib..

[33] Lee J.H., Kim J. (2002). Analysis of sound transmission through periodically stiffened panels by space-harmonic expansion method. J. Sound Vib..

[34] Mathur G.P., Tran B.N., Bolton J.S., Shiau N.M. Sound transmission through stiffened double-panel structures lined with elastic porous materials. Proceedings of the 14th DGLR/AIAA Aeroacoustics Conference.

[35] Xin F.X., Lu T.J. (2011). Analytical modeling of wave propagation in orthogonally rib-stiffened sandwich structures: Sound radiation. Comput. Struct..

[36] Wang J., Lu T.J., Woodhouse J., Langley R.S., Evans J. (2005). Sound transmission through lightweight double-leaf partitions: Theoretical modelling. J. Sound Vib..

[37] Xin F.X., Lu T.J. (2009). Analytical and experimental investigation on transmission loss of clamped double panels: Implication of boundary effects. J. Acoust. Soc. Am..

